# Analysis of the post-incorporation period of health technologies incorporated into the SUS, from 2012 to 2022

**DOI:** 10.11606/s1518-8787.2025059006732

**Published:** 2025-10-24

**Authors:** Nayê Balzan Schneider, Ana Paula Beck Da Silva Etges, Carisi Anne Polanczyk

**Affiliations:** I Universidade Federal do Rio Grande do Sul Programa de Pós-Graduação em Epidemiologia Porto Alegre RS Brazil Universidade Federal do Rio Grande do Sul. Programa de Pós-Graduação em Epidemiologia. Porto Alegre, RS, Brazil; II Instituto de Avaliação de Tecnologia em Saúde Porto Alegre RS Brazil Instituto de Avaliação de Tecnologia em Saúde. Porto Alegre, RS, Brazil

**Keywords:** Brazilian Unified Health System, Health Technology, Access to Health Technologies

## Abstract

**OBJECTIVE:**

To analyze the time elapsed in the post-incorporation process of procedures, orthoses, prostheses, and special materials, with a focus on compliance with the legal deadline of 180 days established for the provision of the technology.

**METHODS:**

The analysis was conducted with procedures and orthoses, prostheses, and special materials recommended for incorporation by the National Commission for the Incorporation of Technologies from 2012 to 2022, and the technology identification code created in the Management System for the Table of Procedures, Medicines, Orthoses, Prostheses and Special Materials of the Brazilian Unified Health System, after incorporation. For the technologies identified, we calculated the median periods (Q1–Q3) of the days elapsed during the incorporation and post-incorporation periods. In addition, the proportion of technologies offered according to the legal deadline was verified, and the influence of variables on the post-incorporation period was analyzed.

**RESULTS:**

Among 41 reports on procedures, orthoses, prostheses, and special selected materials, 79 technologies were analyzed. The coding period, defined as the benchmark for the supply of the technology, had a median of 204 (57–425) days. This period was longer than 180 days in 64% of the technologies assessed. Variables such as the organization group to which the technology belongs, indication of the need to adapt for implementation in the incorporation report, and delay in the evaluation period for incorporation seem to influence the period for providing the technology.

**CONCLUSIONS:**

The effective provision of technologies incorporated into the health system in Brazil has not occurred within the expected period of 180 days in most cases, which limits their accessibility to the population. Anticipating the need for adaptation before implementation, even during incorporation, seems to be a way of reducing the post-incorporation period.

## INTRODUCTION

The opportunity for the population to access a new health technology includes actors engaged in different stages of the process, which determine the technology’s availability and accessibility. Availability refers to the first stages of the technology’s introduction, such as marketing authorization. Accessibility, on the other hand, refers to the subsequent stages that involve actual access to the technologies, influenced, for example, by pricing and subsidies from health systems^1,2^ .

Among the actors involved from the start of the access process are: the industries, which develop the new products; the regulatory bodies, responsible for reviewing efficacy and safety and availability on the market; and the health technology assessment (HTA) bodies, which include economic assessment and efficacy and safety analyses to support the incorporation decision into a given health system. In addition to these, other parties, such as patients and health providers (institutions and health professionals), also play a relevant role in the technologies provided to the population^3^. Therefore, the way each country’s health system organizes the participation of the actors and the steps to be taken directly interferes with the population’s access to the new technologies.

In Brazil, the National Health Surveillance Agency, as the country’s regulatory body, is responsible for authorizing the marketing of a new product, making it available on the market. Once it is available, it is possible to start the process of incorporation into the Brazilian Unified Health System (SUS), in order to guarantee the technology’s accessibility. The incorporation process begins with the filing of the request for incorporation and ends with the publication of the decision in the *Diário Oficial da União* (DOU), which must take place within 270 days (180 days which can be extended for a further 90 days)^4,5^ . The incorporation process has a well-established flow of activities that is transparent to the population and takes place in a standardized way for all technologies, be they medicines, procedures, equipment, or materials^4^.

Next, the process to enable the provision of the incorporated technology begins, referred to here as the post-incorporation period. This stage aims to ensure the effective accessibility of the technology to the population and must take place within a new 180-day period. The complexity of this process, which involves performing different actions depending on the technology in question, poses significant challenges to meeting the stipulated deadline^6^. Among the actions to promote accessibility after incorporation are the agreement on financing and technology acquisition responsibilities, drawing up/updating clinical guidelines, establishing purchase contracts, and creating the technology’s identifying code in the SUS Management System for the Table of Procedures, Medicines, Orthoses, Prostheses and Special Materials (SIGTAP)^9^. The post-incorporation process has no established flow of steps, making the process less transparent to the population.

In the national literature, there is a variety of studies on the incorporation process, whereas the post-incorporation period is less covered. There is also great variety in the types of technologies researched. Analyses generally focus on medicines, while procedures and products are less explored. This difference is also noticeable in the profile of the technologies evaluated and made available to the population^10^. In the recommendations panel of the *Comissão Nacional de Incorporação de Tecnologias no Sistema Único de Saúde* (Conitec - National Commission for the Incorporation of Technologies into the Unified Health System), when analyzing the period between 2012 and 2024, it is possible to see that medicines are the technologies with the highest number of demands assessed and with positive decisions for incorporation (77% and 68%, respectively). Procedures account for 17% of the technologies demanded and 26% of the technologies incorporated, while products account for 6% of the technologies demanded and incorporated^11^. This pattern of incorporation had already been pointed out in the literature, in a publication that evaluated technologies incorporated up to 2019^12^.

Given this context, this study aims to explore the post-incorporation process of procedures and orthoses, prostheses, and special materials (OPMs) within the SUS, identifying the time required to implement their provision, the proportion of technologies offered within the legal timeframe, and the influence of variables on this process.

## METHODS

### Selection of Technologies

This is a retrospective, documentary analysis study, using the DOU, SIGTAP, the Conitec incorporation report, and data from the SUS Outpatient Information System (SIA/SUS) and the SUS Hospital Information System (SIH/SUS) as sources of information.

In order to select the technologies to be included in the analysis, the reports recommending their incorporation on the Conitec website were consulted, using the following selection criteria: reports between 2012 and 2022, evaluating diagnostic procedures, clinical procedures, surgical procedures and OPMs with a favorable recommendation for incorporation. Cases of expanded use were not considered, as they refer to technologies that have already been incorporated. After selecting the reports, each technology was searched for in SIGTAP using terms related to its identification. For technologies not located in SIGTAP, the code number was requested via the Access to Information Act.

The technologies selected included all procedures (group codes 02, 03, and 04, respectively, diagnostic procedures, clinical procedures, and surgical procedures) and OPMs (group codes 07) for which a code was identified in SIGTAP created after incorporation.

### Analysis of the Post-Incorporation Period

Three periods were analyzed, expressed in days elapsed, according to the following definition:

- **Coding period:** Number of days elapsed between the date of publication in the DOU and the date the SIGTAP code was created. The inclusion of the code in SIGTAP indicates that the technology is now part of the SUS management system and can be reported by service providers who are prepared to implement it. As such this period was used to assess whether the technology was made available within the legally mandated 180-day timeframe.
- **Period of use:** Number of days elapsed between the date of SIGTAP code creation and the date of the first recorded use of the technology in the SIA or SIH.
- **Post-incorporation period:** Number of days elapsed between the date of publication in the DOU and the date of the first record in the SIA or SIH (this includes the coding period and the period of use)*.*

### Complementary Analysis

As a complement to the post-incorporation analysis, in order to understand the entire accessibility process, two other periods were evaluated:

- **Incorporation period**: Number of days elapsed between the date the incorporation request was filed and the date the decision was published in the DOU. In other words, it is the period necessary for conducting an assessment of incorporation and reaching a final decision on the incorporation of technology.
- **Access period**: Number of days elapsed between the date of filing of the request for assessment of incorporation and the date of the first recorded use of the technology in the SIA or SIH (this includes the incorporation period and the post-incorporation period).

The format for the date of inclusion of the code in SIGTAP and the date of the first record in SIH and SIA is month/year, so the first day of each month was considered when calculating the periods.

### Statistical Analysis

The periods were analyzed using descriptive statistics, which appear as median (first quartile [Q1] – third quartile [Q3]) and mean (standard deviation [SD]) of days elapsed. In order to analyze the proportion of technologies offered within the mandated timeframe, the number of technologies with a SIGTAP code created within 180 days of the incorporation decision (the *period for coding*) was verified, among the 79 technologies identified.

In addition to the descriptive measures, non-parametric tests (Mann-Whitney or Kruskal-Wallis, as appropriate) were carried out to see if there was an effect of the variables of interest in the *post-incorporation period*. Ten variables were considered, defined on the basis of characteristics with a possible influence on the post-incorporation period: i) year of publication of the incorporation decision in the DOU (first or last 5 years evaluated); ii) delay in the incorporation decision (whether or not there was a delay, considering 270 days); iii) mention of the Clinical Protocols and Therapeutic Guidelines (PCDT)/guideline/protocol for the technology in the Conitec report (whether or not there is mention); iv) mention of the need for adaptation for implementation in the Conitec report (whether or not there is mention of the need); v) need for adaptation for implementation in the Conitec report (whether or not there is a need for adaptation); vi) information on the form of payment/financing/financial transfer in the Conitec report (whether or not there is information); vii) financial impact in the Conitec report (whether it is less or more than 1 million reais); viii) incremental cost-effectiveness ratio in the Conitec report (RCEI; if it is more or less than 50,000 reais); ix) technology group according to SIGTAP (02- diagnostic procedures, 03-clinical procedures, 04-surgical procedures, 07-OPMs); and x) type of financing according to SIGTAP (whether it receives a transfer from Medium and High Complexity or Health Surveillance). In addition, in order to capture differences in the post-incorporation period between the subgroups of technologies defined in SIGTAP, the same statistical tests were performed intra-group. A p-value < 0.05 was considered significant. The statistical analyses were performed in the R language (version 4.2.0) using the RStudio© software (version 2023.03.1 Build 446, Posit Software, PBC) for Windows^13^.

## RESULTS

From 2012 to 2022, 72 reports of procedures and OPMs with a recommendation for incorporation by Conitec were identified. Of these, 31 were not included in the analysis, 13 because they assessed technologies with codes that already existed in SIGTAP, 4 because they referred to technologies with the same SIGTAP code, and 14 because the corresponding codes had not been created (the non-inclusion of these 14 technologies in SIGTAP was confirmed in a consultation with the Ministry of Health – protocol no. 25072.005034/2025-34). Thus, 41 reports were analyzed, which evaluated 79 technologies, corresponding to 79 new codes.

During the period evaluated, the number of technologies approved varied each year, with the lowest number in 2015 (n = 1) and the highest number in 2014 (n = 35). Of the 79 technologies, 31 (39%) are OPMs, such as the pediatric shell-style shower wheelchair and the bone-anchored hearing aid. The remaining 48 (61%) technologies refer to procedures, including diagnostic procedures, such as optical coherence tomography and whole exome sequencing; clinical procedures, such as maintenance of cochlear implant prosthesis and non-cosmetic sclerotherapy for varicose veins in the lower limbs; and surgical procedures, such as type 2 cervical excision and bilateral cochlear implant surgery. Most of the technologies analyzed have medium- and high-complexity funding, and the majority of those requesting their incorporation are public bodies, mostly secretariats of the Ministry of Health and state secretariats. In the analysis of Conitec’s incorporation reports, it was also found that for most of the technologies there is no mention of the PCDT, guideline or protocol of use in which it will be included, nor of the RCEI. However, the form of payment and the technology’s financial impact are provided for most of the technologies analyzed. Considering the 270-day deadline, there was a delay in publishing the incorporation decision for 49% of the technologies evaluated (Table 1).

**Table 1:** Characterization of the 79 technologies evaluated.

Characteristics	Technology (n = 79)	Report (n = 41)
Year of publication of SCTIE ordinance in DOU		
2013	17 (22)	7 (17)
2014	35 (44)	9 (22)
2015	1 (1)	1 (2)
2016	3 (4)	3 (7)
2017	3 (4)	2 (5)
2018	5 (6)	4 (10)
2019	4 (5)	4 (10)
2020	2 (3)	2 (5)
2021	5 (6)	5 (12)
2022	4 (5)	4 (10)
Requester		
Ministry of Health^a^	7 (9)	7 (17)
SVS/MS	7 (9)	7 (17)
SAS/MS	51 (65)	15 (37)
State Secretariat	6 (8)	4 (10)
Municipal office	1 (1)	1 (2)
Medical society	3 (4)	3 (7)
Hospital de Clínicas de Porto Alegre	1 (1)	1 (2)
NI	3 (4)	3 (7)
Does the report mention a PCDT, guideline or protocol for the use of the technology?		
Yes	10 (13)	10 (23)
No	69 (87)	31 (78)
Does implementation require adaptation?		
Yes	7 (8)	7 (17)
No	32 (41)	14 (35)
NI	40 (51)	20 (50)
Does the report indicate the form of payment/transfer?		
Yes	47 (60)	11 (27)
No	32 (40)	30 (73)
Financial impact in 1^st^ year (R$)		
Less than 1 million	17 (21)	10 (24)
More than 1 million	36 (46)	26 (64)
NI	26 (33)	5 (12)
RCEI (R$)		
Less than 50 thousand	13 (17)	11 (27)
More than 50 thousand	3 (4)	4 (10)
NI	62 (78)	25 (61)
NA (cost minimization)	1 (1)	1 (2)
Was there a delay in incorporation?^b^		
Yes	38 (48)	14 (34)
No	41 (52)	27 (66)
Financing		
Medium and High Complexity^c^	72 (91)	NA
Health surveillance	7 (9)	NA
Group		NA
Diagnostic procedures	21 (26)	NA
Clinical procedures	9 (11)	NA
Surgical procedures	18 (23)	NA
Orthoses, prostheses, and special materials	31 (40)	NA

NA: not applicable, characteristics that refer only to technologies. NI: not informed; DOU: *Diário Oficial da União*; RCEI: incremental cost-effectiveness ratio; SVS/MS: Secretariat of Health Surveillance of the Ministry of Health; SAS/MS: Secretariat of Health Care of the Ministry of Health; PCDT: clinical protocol and pharmaceutical guideline; SCTIE: Secretariat of Science, Technology and Strategic Inputs.

Note: data presented as number of technologies or reports and proportion.

^a^ Secretariat of Science, Technology and Strategic Inputs (n = 6) and Intellectual Disability PCDT Elaboration Group (n = 1).

^b^ Decisions published within more than 270 days were considered as delayed. Considering the period of 180 days, without the extension, there is a delay in 65% of the technologies.

^c^ Composed of the Strategic Actions and Compensation Fund component, in which federal financial resources are transferred after the verification of health service production recorded by the respective managers in the Outpatient and Hospital Information Systems; and the Medium and High Complexity Financial Limit component, in which federal financial resources are regularly and automatically transferred from the National Health Fund to the health funds of the states, the Federal District, and municipalities.

There has been a change in the reporting profile over the years, such as the inclusion of the ICER analysis consistently in reports filed from 2017 onwards. In addition to this change, it was found that in the incorporation requests filed until 2014, information on payment/financing/transfer methods was more frequent (59% before 2014 and 7% after 2014) and whether or not there was a need for adaptations (65% before 2014 and 23% after 2014).

For the 79 technologies selected, the *coding period* had a median (Q1–Q3) of 204 (57–425) days and a mean (SD) of 373 (472) days. This period, used to assess the effective provision of the technology, exceeded the legal deadline of 180 days in 64% of the technologies evaluated (Table 2). Among the technologies evaluated, the shortest period was for a clinical procedure, related to genetic counseling (29 days), and the longest was for a diagnostic procedure, the Xpert MTB/RIF test for tuberculosis (2,119 days, almost six years of delay).

**Table 2:** Post-incorporation periods according to technology groups.

Period	Total (n = 79)	Diagnostic procedures (n = 21)	Clinical procedures (n = 09)	Surgical procedures (n = 18)	OPMs (n = 31)
Coding					
Median (Q1–Q3)	204 (57–425)	408 (204–1,021)	59 (29–204)	204 (204–514)	65 (54–235)
Mean (SD)	373 (472)	715 (683)	120 (141)	356 (256)	226 (322)
Technologies with delay; n (%)	51 (64)	16 (76)	3 (33)	17 (94)	15 (48)
Use					
Median (Q1–Q3)	28 (0–304)	31 (0–273)	578 (0–1,068)	213 (30–1,034)	0 (0–0)
Mean (SD)	290 (513)	245 (542)	603 (494)	554 (649)	16 (46)
Post-incorporation^a^					
Median (Q1–Q3)	442 (85–1,023)	782 (285–1,113)	782 (442–1,097)	801 (543–1,238)	54 (54–235)
Mean (SD)	649 (627)	921 (770)	723 (436)	910 (576)	249 (384)

SD: standard deviation; Q1: first quartile; Q3: third quartile; OPM: Orthoses, Prostheses and Special Materials; SIGTAP: Management System for the SUS Table of Procedures, Medicines, and OPM.

^a^ Ten technologies had not been dispensed until the last check in December 2024, although they already had the code in SIGTAP, so 69 technologies were considered in this calculation.

Among the types of technology classified according to the SIGTAP organization group, clinical procedures and OPMs have a median *coding period* that complies with the legal deadline, 59 and 65 days, respectively. Surgical procedures have a median *coding period* of 204 days and account for the highest number of delayed technologies (94%), followed by diagnostic procedures, with a median of 408 days and 76% of technologies delayed (Table 2).

The median *period for use* across all technologies was 28 (0–304) days. The type of technology with the lowest median was OPMs, for which use was recorded a few days after registration in SIGTAP (median 0 [0–0]; mean 16 [46] days). By December 2024, ten incorporated technologies were identified for which no use was recorded in the respective information system after the SIGTAP code was created (six OPMs and four diagnostic procedures). The median time for the entire post-incorporation process was 442 (85–1,023) days. OPMs were the technologies with the shortest *post-incorporation period*, with a median of 54 (54–235) days and a mean of 249 (384) days (Table 2).

**Figure 1:**
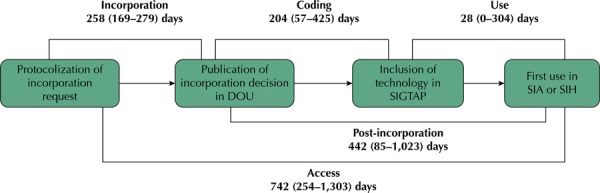
Representation of the periods analyzed for procedures and OPMs. DOU: *Diário Oficial da União*; OPM: orthoses, prostheses, and special materials; SIA: Outpatient Information System; SIH: Hospital Information System; SIGTAP: SUS Management System for the Table of Procedures, Medicines, and OPM.

Note: period shown as median (Q1–Q3) days.

Finally, the *access period* showed the entire process of incorporation and post-incorporation*.* Across all the technologies evaluated, the median for this period is 742 days (254–1,303), i.e., for 50% of these technologies, access to procedures or OPMs in Brazil occurs approximately two years after the request for incorporation. As part of the *access period*, the median *incorporation period* is 258 (169–279) days (Figure 1).

**Figure 2:**
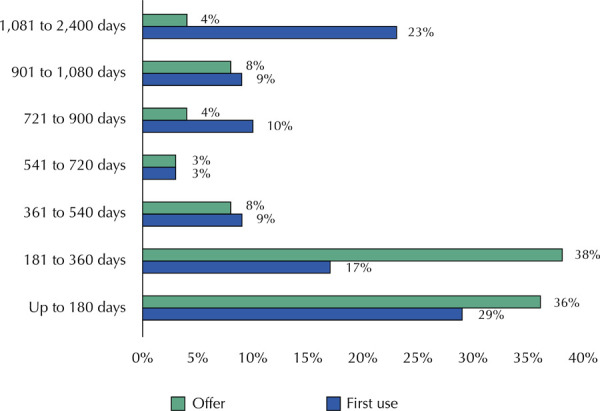
Frequency of offer and first use of the technologies analyzed since publication of their incorporation in the DOU, according to the length of time elapsed.

Note: as offer, we considered the *period for coding:* number of days between the publication of the incorporation in the *Diário Oficial da União* (DOU) and the creation of the code in the Management System for the Table of Procedures and Medicines (SIGTAP). For first use, the *post-incorporation period* was considered: number of days between publication in the DOU and the first record of use in the information system.

The analysis of how frequently technologies are included in SIGTAP and first used, according to elapsed time intervals, shows that most technologies (74%) are incorporated into the management system within 360 days. Regarding actual use, 46% of the technologies had their first dispensing within 360 days, indicating that the majority of technologies had not yet been used by the population during this period (Figure 2).

In the analyses of the *post-incorporation period* carried out according to the variables of interest, there was an influence of the SIGTAP organization group, indication of the need for adaptations for implementation, need for adaptations and delay in incorporation (p < 0.05; Table 3). Among the types of technologies, defined by the group to which they belong in SIGTAP, it was found that the post-incorporation period is significantly longer for any of the procedures compared to OPMs. Significant differences between the technology subgroups within each SIGTAP group were not identified in the procedures analyzed (p > 0.05 for all). Across the OPMs, the comparison would be between the subgroup of technologies related to the surgical act and the subgroup not related to the surgical act; however, this was not carried out because the six OPMs related to the surgical act do not have first-use records.

**Table 3:** Days between the decision to incorporate and the first use of the technology, according to the variables of interest.

Category	n	Median (Q1–Q3)	p-value
Year of publication in DOU			
2013–2017	53	235 (56–1,097)	NS
2018–2022	16	624 (464–926)	
SIGTAP Group			
Diagnostic procedures	17	782 (285–1,113)	< 0.001^a^
Clinical procedures	9	782 (442–1,097)	
Surgical procedures	18	802 (544–1,239)	
Orthoses, prostheses, and special materials	25	54 (54–235)	
Financing			
Medium and high complexity	64	403 (79–1,015)	NS
Health surveillance	05	951 (483–1,113)	
Does the Conitec report mention a PCDT, guideline or protocol?			
Yes	08	384 (234–629)	NS
No	61	442 (59–1,097)	
Does the Conitec report mention the need for adaptation for implementation?			
Yes	37	235 (54–821)	< 0.01
No	32	782 (370–1,345)	
Is adaptation necessary for implementation?			< 0.05
Yes	6	869 (606–988)	
No	31	212 (54–352)	
Does the Conitec report indicate payment method?			
Yes	43	235 (54–1,097)	NS
No	26	504 (244–916)	
Financial impact (1st year)			
Less than R$ 1 million	14	263 (235–464)	NS
Greater than R$ 1 million	34	127 (54–581)	
RCEI			
Less than R$ 50 thousand	9	596 (483–951)	NS
Greater than R$ 50 thousand	3	821 (737–866)	
Was there a delay in the Conitec evaluation?			
Yes	31	782 (420–1.496)	< 0.001
No	38	129 (54–533)	

Conitec: National Commission for the Incorporation of Technologies; DOU: *Diário Oficial da União*; NS: not significant; PCDT: clinical protocol and pharmaceutical guideline; RCEI: incremental cost-effectiveness ratio, SIGTAP: Management System for the SUS Table of Procedures, Medicines, and OPM.

Note: The Kruskal-Wallis test was used to analyze the SIGTAP Group variable, and the Mann-Whitney test for the others.

^a^ After Bonferroni correction, p-value < 0.05 in the comparison of all groups of procedures in relation to the group Orthoses, prostheses, and special materials.

## DISCUSSION

This is the first study in Brazil to address the period required for accessibility of SUS technologies classified as procedures and OPMs. We analyzed the process with a focus on the period after the technologies were incorporated, in order to identify the number of days that had elapsed and the proportion of technologies provided within the legal timeframe. Variables with a possible impact on the *post-incorporation period* were also analyzed.

The data obtained indicate that, considering the *coding period* (the interval between publication in the DOU and the inclusion of the technology in the SIGTAP table) as the benchmark for making the technology available, 64% of the evaluated technologies were delayed relative to the 180-day deadline, and 50% of the technologies are included in SIGTAP within 204 (57–425) days. This high proportion of technologies provided after the established deadline has also been identified for medicines^6^. National studies indicate a delay of 65% for medicines in the Specialized Component of Pharmaceutical Assistance (CEAF) and 100% for arthritis medicines^6,8^.

After inclusion in SIGTAP, the first use occurred after 28 (0–304) days in 50% of the technologies, resulting in a total post-incorporation period with a median of 442 (85–1,023) days. The *coding period*, as well as the *period for use* (period between inclusion in SIGTAP and first use), varied greatly across the types of technologies analyzed. It was found that, after being included in SIGTAP, diagnostic procedures and OPMs have a shorter period until first use compared to clinical and surgical procedures. The long period until the first use of clinical procedures (median of 578 [0–1,068] days) and surgical procedures (median of 213 [30–1,034] days) indicates that these procedures still need actions to be completed before they can be used. Possible actions include publishing PCDTs, institutional adjustments, price negotiations, budget adjustments, and establishing contracts. Although it is not clear when each of these actions takes place (whether before or after inclusion in SIGTAP), it is known that they occur after the decision to incorporate, which allows for the analysis of some factors possibly related to the post-incorporation period as a whole.

There was variation in the number of days elapsed during the *post-incorporation period* (period between the decision to incorporate and first use) for the groups of technologies evaluated. Surgical procedures had the longest period (median 802 [544–1,239]), and a significantly greater difference was observed when comparing any of the procedures relative to OPMs. These differences observed between the types of technologies may be related to variations in their complexity. While OPMs include individually used supplies for health care, medical procedures involve direct actions on the patient, often requiring specialized technical knowledge and specific infrastructure^14,15^.

In addition to the SIGTAP organization group, the results indicate that factors such as the indication of the need for adjustments for technology implementation in the Conitec report, as well as the need for adaptations and delays in the assessment for incorporation decisions, influence the *post-incorporation period*. There was a significantly longer period for technologies in which the need for adaptations is not mentioned (*versus* being mentioned), in which adaptations are necessary (*versus* not being necessary) and for those in which there was a delay in incorporation (*versus* no delay). Indicating the need for adjustments, whether to the infrastructure of the institutions or to the professionals involved with the technology, in the incorporation assessment report, is important because these are actions that require planning, and if they are already defined during the incorporation process, they allow for a shorter post-incorporation period. Cases that require adjustment also require considerable time to complete, since in addition to training the technical team responsible, there is a learning curve that varies according to the technology’s complexity^15^.

With regard to the longer period identified for technologies that have already had a delay in the decision to incorporate them, it is understood that there are complicating factors in the incorporation assessment that extend to the post-incorporation period. One of these is in relation to the availability of quality scientific evidence, which can influence both the decision on incorporation and the drafting of PCDTs, which takes place in the post-incorporation period^16^. It also indicates the complexity of the whole HTA process for procedures and OPMs, as these are technologies with specific characteristics, which differ from the evaluation of medicines^17,18^.

In order to improve the process of implementing technologies, some recommendations for change have been made, including agreeing on responsibilities for financing and acquiring technology, verifying structural requirements for the implementation of technology use, and developing/updating clinical guidelines^9^. The data from this study regarding adjustment variables corroborate the importance of anticipating the requirements for technology use.

Among the incorporation reports analyzed, 14 did not have the technologies and corresponding codes identified in SIGTAP, and 53% of these technologies were incorporated after 2020. This indicates that, although approved for incorporation, these technologies have not yet been made available on a regular basis in the SUS, i.e., they are not being offered to the population. These technologies were not included in the analysis of this study, but if they were, they would increase the proportion of delayed procedures and OPMs to 70%.

The limitations of this study include the fact that only publicly available online data sources were used, regarding the national organization for access to technologies, without taking into account local particularities across states and municipalities. Additionally, since there is no transparency and predictability in the flow of activities for the post-incorporation process of the technologies analyzed, the discussion of each period also has limitations. Here, we consider that the creation of the SIGTAP code is a final step in the process that will enable the use of the technology and, for this reason, the coding period was defined as a benchmark for the effective provision of the technology. Brazilian legislation, in Decree 7.646/2011, defines that an incorporated technology must be offered to the population within 180 days, but it does not define the benchmark that will indicate this offer^19^. We understand that the existence of the code does not guarantee that all the stages of the post-incorporation process have been completed, and this is verified by the difference between the *coding period* and the *post-incorporation period*. However, the consideration of another benchmark, such as the date of first use to verify compliance with the legal deadline, raises questions such as the need for the technology to be used, which may not represent the period in which it was already available but did not need to be used. Considering these factors, we decided to choose the shortest period possible to be measured in order to verify compliance with the timeframe.

Measuring the periods and the proportion of procedures and OPMs not provided within the legal timeframe is presented in this study as a first step towards understanding the current scenario for the accessibility of these technologies in the country, which is necessary if better results are to be achieved in terms of post-incorporation efficiency^20^. It is understood that speeding up the provision of new technologies is relevant for both patients and health systems, and that a better understanding of the post-incorporation process can drive improvement actions.
