# Supplementary material for: Analysis of the post-incorporation period of health technologies incorporated into the SUS, from 2012 to 2022
Source: Rev Saude Publica. 2025 Oct 24;59:e29. doi: 10.11606/s1518-8787.2025059006732 (PMC12666786; doi:10.11606/s1518-8787.2025059006732)
Supplement: Translation

# Translation: Análise do período pós-incorporação de tecnologias de saúde incorporadas ao SUS entre 2012 e 2022

**Authors:** Nayê Balzan Schneider, Ana Paula Beck Da Silva Etges, Carisi Anne Polanczyk

**Affiliations:** I Brasil Universidade Federal do Rio Grande do Sul. Programa de Pós-Graduação em Epidemiologia. Porto Alegre, RS, Brasil; II Brasil Instituto de Avaliação de Tecnologia em Saúde. Porto Alegre, RS, Brasil

**Keywords:** Sistema Único de Saúde, Tecnologia em Saúde, Acesso a Tecnologias em Saúde

## Abstract

**OBJETIVO:**

Analisar o período transcorrido no processo pós-incorporação de procedimentos, órteses, próteses e materiais especiais, observando o cumprimento ao prazo legal de 180 dias estabelecido para a oferta da tecnologia.

**MÉTODOS:**

A análise foi realizada com procedimentos e órteses, próteses e materiais especiais com recomendação de incorporação pela Comissão Nacional de Incorporação de Tecnologias entre 2012 e 2022, e código de identificação da tecnologia criado no Sistema de Gerenciamento da Tabela de Procedimentos, Medicamentos, Órteses, Próteses e Materiais Especiais do SUS após a incorporação. Para as tecnologias identificadas, foram calculados períodos que informam a mediana (Q1–Q3) dos dias transcorridos nos períodos de incorporação e pós-incorporação. Adicionalmente, foi verificada a proporção de tecnologias ofertadas conforme prazo legal, e analisada a influência de variáveis no período pós-incorporação.

**RESULTADOS:**

Entre 41 relatórios de procedimentos, órteses, próteses e materiais especiais selecionados, 79 tecnologias foram analisadas. O período para codificação, definido como marco da oferta da tecnologia, apresenta mediana de 204 (57–425) dias. Esse período foi maior que 180 dias em 64% das tecnologias avaliadas. Variáveis como o grupo de organização a que a tecnologia pertence, indicação da necessidade de adequação para implementação no relatório de incorporação e atraso no período de avaliação para incorporação parecem influenciar o período para oferta da tecnologia.

**CONCLUSÕES:**

A efetiva oferta das tecnologias incorporadas no sistema de saúde no Brasil não tem ocorrido dentro do período previsto de 180 dias na maioria dos casos, o que limita seu oferecimento à população. A previsão da necessidade de adequação para implementação, ainda durante a incorporação, parece ser uma forma de diminuir o período pós-incorporação.

## INTRODUÇÃO

A oportunidade de acesso da população a uma nova tecnologia de saúde inclui atores com envolvimento em diferentes etapas do processo, as quais definem a disponibilidade e a acessibilidade à tecnologia. A disponibilidade refere-se às primeiras etapas do processo de oferta de uma nova tecnologia, proporcionada, por exemplo, pela autorização de comercialização. Já a acessibilidade refere-se às etapas seguintes, que envolvem o real acesso às tecnologias, influenciadas, por exemplo, por questões de preço e subsídios dos sistemas de saúde^1,2^.

Entre os atores envolvidos desde o início do processo de acesso estão: as indústrias, que desenvolvem os novos produtos; os órgãos reguladores, responsáveis pela revisão da eficácia e segurança e pela disponibilização ao mercado; e os órgãos de avaliação de tecnologias em saúde (ATS), que incluem a avaliação econômica e as análises de eficácia e segurança para embasar a decisão de incorporação em determinado sistema de saúde. Além destas, outras partes, como pacientes e provedores de saúde (instituições e profissionais de saúde), também desempenham papel relevante nas tecnologias ofertadas à população^3^. Sendo assim, a forma como o sistema de saúde de cada país organiza a participação dos atores e das etapas a serem percorridas interfere diretamente no acesso da população às novas tecnologias.

No Brasil, a Agência Nacional de Vigilância Sanitária, por ser o órgão regulador do país, é responsável por autorizar a comercialização de um novo produto, tornando-o disponível no mercado. Após a disponibilização, é possível iniciar o processo de incorporação no Sistema Único de Saúde (SUS), a fim de garantir a acessibilidade da tecnologia. O processo de incorporação inicia com a protocolização do pedido de incorporação e se encerra com a publicação da decisão no Diário Oficial da União (DOU), o que deve ocorrer dentro de 270 dias (180 dias que podem ser prorrogados por mais 90 dias)^4,5^. O processo de incorporação possui um fluxo de atividades bem estabelecido e transparente à população, ocorrendo de maneira padronizada para todas as tecnologias, sejam elas medicamentos, procedimentos, equipamentos ou materiais^4^.

Em seguida, tem início o processo para viabilizar a oferta da tecnologia incorporada, aqui identificado como pós-incorporação. Essa etapa visa promover a efetivação da oferta à população e deve ocorrer dentro de um novo período de 180 dias. A complexidade desse processo, que envolve a execução de diferentes ações a depender da tecnologia em questão, confere desafios importantes para cumprimento do prazo estipulado^6^. Entre as ações para promover a efetivação de oferta da tecnologia incorporada estão a pactuação das responsabilidades de financiamento e de aquisição da tecnologia, a elaboração/atualização das diretrizes clínicas, estabelecimento de contratos de compra e a criação do código identificador da tecnologia no Sistema de Gerenciamento da Tabela de Procedimentos, Medicamentos, Órteses, Próteses e Materiais Especiais (OPM) do SUS (SIGTAP)^9^. O processo pós-incorporação não tem um fluxo de etapas estabelecido, tornando o processo menos transparente à população.

Na literatura nacional, há uma variedade de trabalhos sobre o processo de incorporação, enquanto o período pós-incorporação é menos abordado. Ainda há grande variedade entre os tipos de tecnologias pesquisadas. As análises geralmente são voltadas aos medicamentos, sendo procedimentos e produtos menos explorados. Essa diferença é perceptível também no perfil das tecnologias avaliadas e disponibilizadas à população^10^. No painel de recomendações da Comissão Nacional de Incorporação de Tecnologias no Sistema Único de Saúde (Conitec), ao analisar o período entre 2012 e 2024, é possível verificar que medicamentos são as tecnologias com maior número de demandas avaliadas e com decisões positivas de incorporação (77% e 68%, respectivamente). Procedimentos correspondem a 17% das tecnologias demandadas e a 26% das tecnologias incorporadas, enquanto produtos correspondem a 6% das tecnologias demandadas e das incorporadas^11^. Esse padrão de incorporação já havia sido apontado na literatura, em publicação que avaliou tecnologias incorporadas até 2019^12^.

Diante desse cenário, este estudo busca explorar o processo pós-incorporação de procedimentos e OPMs no SUS, identificando o período necessário para efetivação da oferta, a proporção de tecnologias ofertadas dentro do prazo legal e a influência de variáveis neste processo.

## MÉTODOS

### Seleção das Tecnologias

Este é um estudo retrospectivo, de análise documental, tendo como fonte de informação o DOU, o SIGTAP, o relatório de incorporação da Conitec e dados do Sistema de Informações Ambulatoriais do SUS (SIA/SUS) e Sistema de Informações Hospitalares do SUS (SIH/SUS).

Para seleção das tecnologias a serem incluídas na análise, foram consultados os relatórios de recomendação de incorporação no site da Conitec, seguindo os seguintes critérios de seleção: relatórios entre 2012 e 2022, com avaliação de procedimentos com finalidade diagnóstica, procedimentos clínicos, procedimentos cirúrgicos e OPMs com recomendação favorável à incorporação. Casos de ampliação de uso não foram considerados, pois se referem a tecnologias já incorporadas. Após a seleção dos relatórios, cada tecnologia foi pesquisada no SIGTAP a partir de termos relacionados a sua identificação. Para as tecnologias não localizadas no SIGTAP, solicitou-se o número do código via Lei de acesso à informação.

As tecnologias selecionadas incluíram todos os procedimentos (códigos de grupo 02, 03 e 04, respectivamente, procedimentos com finalidade diagnóstica, procedimentos clínicos e procedimentos cirúrgicos) e OPMs (códigos de grupo 07), para as quais foi identificado código no SIGTAP criado após a incorporação.

### Análise do Período Pós-Incorporação

Foram analisados três períodos, expressos em dias transcorridos, conforme a seguinte definição:

- **Período para codificação:** dias transcorridos entre a data da publicação no DOU e data de criação do código SIGTAP. A inserção do código no SIGTAP sinaliza que a tecnologia está no sistema de gerenciamento do SUS e seu uso pode ser registrado pelo prestador de serviço que estiver preparado para implementá-la. Sendo assim, este foi o período considerado para verificar a efetivação da oferta da tecnologia quanto ao cumprimento do prazo legal de 180 dias.
- **Período para uso:** dias transcorridos entre a data de criação do código SIGTAP e data do primeiro registro de uso da tecnologia no SIA ou SIH.
- **Período pós-incorporação:** dias transcorridos entre a data da publicação no DOU e data do primeiro registro no SIA ou SIH (engloba o período para codificação e o período para uso)*.*

### Análise Complementar

De forma complementar à análise pós-incorporação, para compreensão de todo processo de acessibilidade, outros dois períodos foram avaliados:

- **Período para incorporação**: dias transcorridos entre a data da protocolização do pedido de incorporação e data da publicação da decisão no DOU. Ou seja, o período necessário para avaliação da incorporação pela Conitec e decisão final sobre a incorporação da tecnologia.
- **Período para acesso**: dias transcorridos entre a data da protocolização do pedido de avaliação da incorporação e data do primeiro registro de uso da tecnologia no SIA ou SIH (engloba o *período para incorporação* e o *período pós-incorporação*).

O formato de data de inclusão do código no SIGTAP e data do primeiro registro no SIH e SIA é de mês/ano, assim, foi considerado o dia primeiro de cada mês para os cálculos dos períodos.

### Análise Estatística

Os períodos foram analisados por estatística descritiva, apresentados como mediana (primeiro quartil [Q1] – terceiro quartil [Q3]) e média (desvio padrão [DP]) de dias transcorridos. Para análise da proporção de tecnologias ofertadas dentro do período legal, verificou-se o número de tecnologias com código SIGTAP criado em até 180 dias após a decisão de incorporação (o *período para codificação*), entre as 79 tecnologias identificadas.

Além das medidas descritivas, foram realizados testes não paramétricos (Mann-Whitney ou Kruskal-Wallis, conforme apropriado) para verificar se há efeito das variáveis de interesse no *período pós-incorporação*. Foram consideradas dez variáveis, definidas a partir de características com possível influência no *período pós-incorporação*: i) ano de publicação da decisão de incorporação no DOU (primeiros ou últimos 5 anos avaliados); ii) atraso na decisão de incorporação (se houve ou não atraso, considerando 270 dias); iii) menção ao Protocolos Clínicos e Diretrizes Terapêuticas (PCDT)/diretriz/protocolo de uso para a tecnologia no relatório da Conitec (se há ou não menção); iv) menção da necessidade de adequação para implementação no relatório da Conitec (se há ou não menção sobre a necessidade); v) necessidade de adequação para implementação no relatório da Conitec (se há ou não necessidade de adequação); vi) informação sobre a forma de pagamento/financiamento/repasse financeiro no relatório da Conitec (se há ou não a informação); vii) impacto financeiro do relatório da Conitec (se é menor ou maior que 1 milhão de reais); viii) razão de custo-efetividade incremental do relatório da Conitec (RCEI; se é maior ou menor que 50 mil reais); ix) grupo de tecnologia conforme SIGTAP (02-procedimentos com finalidade diagnóstica, 03-procedimentos clínicos, 04-procedimentos cirúrgicos, 07-OPMs); e x) tipo de financiamento conforme SIGTAP (se recebe repasse da Média e Alta Complexidade ou da Vigilância em saúde). Adicionalmente, a fim de captar diferença no *período pós-incorporação* entre os subgrupos das tecnologias definidos no SIGTAP, os mesmos testes estatísticos foram realizados intragrupo. O valor-p < 0,05 foi considerado significante. As análises estatísticas foram realizadas em linguagem R (versão 4.2.0) por meio do software RStudio© (versão 2023.03.1 Build 446, Posit Software,PBC) para Windows^13^.

## RESULTADOS

Entre 2012 e 2022 foram identificados 72 relatórios de procedimentos e OPMs com recomendação de incorporação pela Conitec. Entre estes, 31 não foram incluídos nas análises, 13 deles por avaliar tecnologias com códigos já existentes no SIGTAP, quatro por referirem-se a tecnologias com o mesmo código SIGTAP, e 14 por não terem sido criados os códigos correspondentes (a não inclusão destas 14 tecnologias no SIGTAP foi confirmada em consulta ao Ministério da Saúde - protocolo nº25072.005034/2025-34). Dessa forma, foram analisados 41 relatórios, os quais avaliaram 79 tecnologias, correspondentes a 79 novos códigos.

No período avaliado, o número de tecnologias aprovadas é variável a cada ano, sendo o menor número verificado em 2015 (n = 1) e o maior número em 2014 (n = 35). Entre as 79 tecnologias, 31 (39%) são OPMs, como a cadeira de rodas para banho em concha infantil e a prótese auditiva ancorada no osso. As demais 48 (61%) tecnologias referem-se a procedimentos, incluindo procedimentos com finalidade diagnóstica, como a tomografia de coerência óptica e o sequenciamento completo do exoma; procedimentos clínicos, como manutenção da prótese de implante coclear e tratamento esclerosante não estético de varizes dos membros inferiores; e procedimentos cirúrgicos, como excisão tipo 2 do colo uterino e cirurgia de implante coclear bilateral. A maioria das tecnologias analisadas possui financiamento de média e alta complexidade, e entre os demandantes dos pedidos de incorporação, a maioria é composta por órgãos públicos, majoritariamente secretarias do Ministério da Saúde e secretarias estaduais. Na análise dos relatórios de incorporação da Conitec, foi verificado ainda que para a maioria das tecnologias não há menção sobre o PCDT, diretriz ou protocolo de uso em que será inserida, assim como sobre a RCEI. Já a forma de pagamento e o impacto financeiro da tecnologia são informados para a maior parte das tecnologias analisadas. Considerando o prazo de 270 dias, houve atraso na publicação da decisão de incorporação de 49% das tecnologias avaliadas (Tabela 1).

**Tabela 1:** Caracterização das 79 tecnologias avaliadas.

Característica	Tecnologia (n = 79)	Relatório (n = 41)
Ano de publicação da portaria SCTIE no DOU		
2013	17 (22)	7 (17)
2014	35 (44)	9 (22)
2015	1 (1)	1 (2)
2016	3 (4)	3 (7)
2017	3 (4)	2 (5)
2018	5 (6)	4 (10)
2019	4 (5)	4 (10)
2020	2 (3)	2 (5)
2021	5 (6)	5 (12)
2022	4 (5)	4 (10)
Demandante		
Ministério da Saúde^a^	7 (9)	7 (17)
SVS/MS	7 (9)	7 (17)
SAS/MS	51 (65)	15 (37)
Secretaria estadual	6 (8)	4 (10)
Secretaria municipal	1 (1)	1 (2)
Sociedade médica	3 (4)	3 (7)
Hospital de Clínicas de Porto Alegre	1 (1)	1 (2)
NI	3 (4)	3 (7)
No relatório é mencionado PCDT, diretriz ou protocolo de utilização da tecnologia?		
Sim	10 (13)	10 (23)
Não	69 (87)	31 (78)
A implementação exige adequação?		
Sim	7 (8)	7 (17)
Não	32 (41)	14 (35)
NI	40 (51)	20 (50)
O relatório indica a forma de pagamento/repasse?		
Sim	47 (60)	11 (27)
Não	32 (40)	30 (73)
Impacto financeiro no 1º ano (R$)		
Menor que 1 milhão	17 (21)	10 (24)
Maior que 1 milhão	36 (46)	26 (64)
NI	26 (33)	5 (12)
RCEI (R$)		
Menor que 50 mil	13 (17)	11 (27)
Maior que 50 mil	3 (4)	4 (10)
NI	62 (78)	25 (61)
NA (custo minimização)	1 (1)	1 (2)
Houve atraso na incorporação?^b^		
Sim	38 (48)	14 (34)
Não	41 (52)	27 (66)
Financiamento		
Média e Alta Complexidade^c^	72 (91)	NA
Vigilância em saúde	7 (9)	NA
Grupo		NA
Procedimentos com finalidade diagnóstica	21 (26)	NA
Procedimentos clínicos	9 (11)	NA
Procedimentos cirúrgicos	18 (23)	NA
Órteses, próteses e materiais especiais	31 (40)	NA

NA: não aplicável, características que se referem somente às tecnologias. NI: não informado; DOU: Diário Oficial da União; RCEI: razão de custo-efetividade incremental; SVS/MS: Secretaria de Vigilância em Saúde do Ministério da Saúde; SAS/MS: Secretaria de Atenção à Saúde do Ministério da Saúde; PCDT: protocolo clínico e diretriz farmacêutica; SCTIE: Secretaria de Ciência, Tecnologia e Insumos Estratégicos.

Nota: dados apresentados como número de tecnologias ou relatórios e proporção.

^a^ Secretaria de Ciência, Tecnologia e Insumos Estratégicos (n = 6) e Grupo Elaborador do PCDT de Deficiência Intelectual (n = 1).

^b^ Considerou-se como atraso as decisões publicadas em mais de 270 dias. Considerando o tempo de 180 dias, sem a prorrogação, é observado atraso em 65% das tecnologias.

^c^ Formado pelo componente Fundo de Ações Estratégicas e Compensação, no qual os recursos financeiros federais são transferidos após a apuração da produção dos estabelecimentos de saúde registrados pelos respectivos gestores nos Sistemas de Informação Ambulatorial e Hospitalar; e pelo componente Limite Financeiro da Média e Alta Complexidade, no qual os recursos financeiros federais são transferidos do Fundo Nacional de Saúde de forma regular e automática aos fundos de saúde dos estados, Distrito Federal e municípios.

Observou-se mudança no perfil de elaboração dos relatórios ao longo dos anos, como na inclusão da análise da RCEI de forma consistente em relatórios protocolados a partir de 2017. Além dessa mudança, foi verificado que nos pedidos de incorporação protocolados até 2014 eram mais frequentes as informações sobre forma de pagamento/financiamento/repasse (59% antes de 2014 e 7% após 2014) e se havia ou não necessidade de adequação (65% antes de 2014 e 23% após 2014).

Para as 79 tecnologias selecionadas, o *período para codificação* apresentou mediana (Q1–Q3) de 204 (57–425) dias e média (DP) de 373 (472) dias. Esse período, considerado para análise da efetivação da oferta das tecnologias, foi maior que o prazo legal de 180 dias em 64% das tecnologias avaliadas (Tabela 2). Entre as tecnologias avaliadas, o menor prazo verificado foi de um procedimento clínico, relacionado ao aconselhamento genético (29 dias), e o maior prazo foi de um procedimento com finalidade diagnóstica, o teste Xpert MTB/RIF para tuberculose (2.119 dias, quase seis anos de atraso).

**Tabela 2:** Períodos pós-incorporação de acordo com os grupos de tecnologias.

Período para	Total (n = 79)	Procedimentos diagnósticos (n = 21)	Procedimentos clínicos (n = 09)	Procedimentos cirúrgicos (n = 18)	OPMs (n = 31)
Codificação					
Mediana (Q1–Q3)	204 (57–425)	408 (204–1.021)	59 (29–204)	204 (204–514)	65 (54–235)
Média (DP)	373 (472)	715 (683)	120 (141)	356 (256)	226 (322)
Tecnologias com atraso; n (%)	51 (64)	16 (76)	3 (33)	17 (94)	15 (48)
Uso^a^					
Mediana (Q1–Q3)	28 (0–304)	31 (0–273)	578 (0–1.068)	213 (30–1.034)	0 (0–0)
Média (DP)	290 (513)	245 (542)	603 (494)	554 (649)	16 (46)
Pós-incorporação^a^					
Mediana (Q1–Q3)	442 (85–1.023)	782 (285–1.113)	782 (442–1.097)	801 (543–1.238)	54 (54–235)
Média (DP)	649 (627)	921 (770)	723 (436)	910 (576)	249 (384)

DP: desvio padrão; Q1: primeiro quartil; Q3: terceiro quartil; OPM: Órteses, Próteses e Materiais Especiais; SIGTAP: Sistema de Gerenciamento da Tabela de Procedimentos, Medicamentos e OPM do SUS.

^a^ Dez tecnologias não tiveram dispensação até a última verificação, em dezembro de 2024, embora já tenham o código no SIGTAP, portanto, foram consideradas 69 tecnologias neste cálculo.

Entre os tipos de tecnologias classificadas conforme grupo de organização do SIGTAP, os procedimentos clínicos e OPMs apresentam mediana do *período para codificação* que cumpre o prazo legal, respectivamente, 59 e 65 dias. Os procedimentos cirúrgicos apresentam mediana de 204 dias no *período para codificação*, e são os que apresentam o maior número de tecnologias em atraso, 94%; seguido dos procedimentos com finalidade diagnóstica, com mediana de 408 dias e 76% das tecnologias em atraso (Tabela 2).

A mediana do *período para uso* entre todas as tecnologias foi de 28 (0–304) dias. O tipo de tecnologia com menor mediana foram as OPMs, para as quais o uso foi registrado poucos dias após o registro no SIGTAP (mediana de 0 [0–0]; média de 16 [46] dias). Até dezembro de 2024, foram identificadas dez tecnologias incorporadas para as quais não foram registrados uso no respectivo sistema de informação após criação do código SIGTAP (seis OPMs e quatro procedimentos diagnósticos). Em relação a todo processo pós-incorporação, verificou-se a mediana de 442 (85–1023) dias. OPMs foram as tecnologias com menor *período pós-incorporação*, apresentando mediana de 54 (54–235) dias e média de 249 (384) dias (Tabela 2).

Por fim, no *período para acesso* verificou-se a totalidade do processo de incorporação e pós-incorporação*.* Entre todas as tecnologias avaliadas, a mediana desse período é de 742 dias (254–1.303), ou seja, para 50% dessas tecnologias, o acesso à procedimentos ou OPMs no Brasil ocorre aproximadamente dois anos após o pedido de incorporação. Como parte do *período para acesso*, a mediana do *período para incorporação* é de 258 (169–279) dias (Figura 1).

**Figura 1:** Representação dos períodos analisados para procedimentos e OPMs. DOU: Diário Oficial da União; OPM: órteses, próteses e materiais especiais; SIA: Sistema de Informação Ambulatorial; SIH: Sistema de Informação Hospitalar; SIGTAP: Sistema de Gerenciamento da Tabela de Procedimentos, Medicamentos e OPM do SUS.

Nota: Período apresentado como mediana (Q1–Q3) de dias.

A análise da frequência com que ocorre a inclusão da tecnologia no SIGTAP e seu primeiro uso, conforme a faixa de tempo transcorrido, demonstra que a maior parte das tecnologias (74%) está no sistema de gerenciamento em até 360 dias. Em relação ao uso, observa-se que em 360 dias houve a primeira dispensação de 46% das tecnologias, indicando que a maior parte das tecnologias ainda não foi utilizada pela população nesse período (Figura 2).

**Figura 2:** Frequência em que ocorreu a oferta e primeiro uso das tecnologias analisadas desde a publicação da incorporação no DOU, conforme a faixa de tempo transcorrido.

Nota: como oferta considerou-se o *Período para codificação:* dias entre a publicação da incorporação no Diário Oficial da União (DOU) e a criação do código no Sistema de Gerenciamento da Tabela de Procedimentos, Medicamentos. Para primeiro uso considerou-se o *Período pós-incorporação*: dias entre a publicação no DOU e o primeiro registro de uso no sistema de informação.

Nas análises do *período pós-incorporação* realizadas de acordo com as variáveis de interesse, verificou-se influência do grupo de organização do SIGTAP, indicação sobre necessidade de adequação para implementação, necessidade de adequação e atraso na incorporação (p < 0,05; Tabela 3). Entre os tipos de tecnologias, definidas pelo grupo a que pertencem no SIGTAP, verificou-se que o período pós-incorporação é significativamente maior para qualquer um dos procedimentos em comparação às OPMs. Diferenças significativas entre os sugbrupos de tecnologias dentro de cada grupo SIGTAP não foram identificadas entre os procedimentos analisados (p > 0,05 para todos). Entre as OPMs, a comparação seria entre o subgrupo de tecnologias relacionados ao ato cirúrgico e o subgrupo não relacionado ao ato cirúrgico, porém, ela não foi realizada pois os seis OPMs relacionados ao ato cirúrgico não possuem registro de primeiro uso.

**Tabela 3:** Dias entre a decisão de incorporação e o primeiro uso da tecnologia, de acordo com as variáveis de interesse.

Categoria	n	Mediana (Q1–Q3)	Valor-p
Ano de publicação no DOU			
2013–2017	53	235 (56–1.097)	NS
2018–2022	16	624 (464–926)	
Grupo SIGTAP			
Procedimentos com finalidade diagnóstica	17	782 (285–1.113)	< 0,001^a^
Procedimentos clínicos	9	782 (442–1.097)	
Procedimentos cirúrgicos	18	802 (544–1.239)	
Órteses, próteses e materiais especiais	25	54 (54–235)	
Financiamento			
Média e alta complexidade	64	403 (79–1.015)	NS
Vigilância em saúde	05	951 (483–1.113)	
O relatório da Conitec menciona PCDT, diretriz ou protocolo?			
Sim	08	384 (234–629)	NS
Não	61	442 (59–1.097)	
O relatório da Conitec menciona necessidade de adequação para implementação?			
Sim	37	235 (54–821)	< 0,01
Não	32	782 (370–1.345)	
É necessária adequação para implementação?			< 0,05
Sim	6	869 (606–988)	
Não	31	212 (54–352)	
O relatório da Conitec indica forma de pagamento?			
Sim	43	235 (54–1.097)	NS
Não	26	504 (244–916)	
Impacto financeiro (1º ano)			
Menor que R$ 1 milhão	14	263 (235–464)	NS
Maior que R$ 1 milhão	34	127 (54–581)	
RCEI			
Menor que R$ 50 mil	9	596 (483–951)	NS
Maior que R$ 50 mil	3	821 (737–866)	
Houve atraso na avaliação Conitec?			
Sim	31	782 (420–1.496)	< 0,001
Não	38	129 (54–533)	

Conitec: Comissão Nacional de Incorporação de Tecnologias; DOU: Diário Oficial da União; NS: não significativo; PCDT: protocolo clínico e diretriz farmacêutica; RCEI: razão custo-efetividade incremental, SIGTAP: Sistema de Gerenciamento da Tabela de Procedimentos, Medicamentos e OPM do SUS.

Nota: O teste Kruskal-Wallis foi utilizado para análise da variável Grupo SIGTAP, e o teste Mann-Whitney para as demais.

^a^ Após correção de Bonferroni, valor-p < 0,05 na comparação de todos os grupos de procedimentos em relação ao grupo Órteses, próteses e materiais especiais.

## DISCUSSÃO

Este é o primeiro estudo no Brasil a abordar o período necessário para acessibilidade de tecnologias do SUS classificadas como procedimentos e OPMs. Analisamos o processo com enfoque no período pós-incorporação das tecnologias, a fim de identificar os dias transcorridos, e a proporção de tecnologias ofertadas dentro do prazo legal. Nesse sentido, também foram analisadas variáveis com possível impacto no *período pós-incorporação*.

Os dados obtidos indicam que, considerando o *período de codificação* (intervalo entre a publicação no DOU e a inclusão da tecnologia na tabela do SIGTAP) como marco da efetivação da oferta, 64% das tecnologias avaliadas apresentaram atraso em relação ao prazo para oferta, estabelecido em 180 dias, sendo que 50% delas são inseridas no SIGTAP em até 204 (57–425) dias. Essa alta proporção de tecnologias ofertadas após o prazo estabelecido também já foi identificada para medicamentos^6^. Estudos nacionais indicam atraso em 65% dos medicamentos do Componente Especializado de Assistência Farmacêutica (CEAF) e de 100% em medicamentos para artrite^6,8^.

Após a inclusão no SIGTAP, o primeiro uso ocorreu após 28 (0–304) dias em 50% das tecnologias, resultando em um período total de pós-incorporação com mediana de 442 (85–1.023) dias. O *período de codificação*, assim como o *período para uso* (período entre inclusão no SIGTAP e primeiro uso), apresentam grande variação entre os tipos de tecnologias analisadas. Verificou-se que, após estarem no SIGTAP, procedimentos diagnósticos e OPMs têm menor período até primeiro uso comparado a procedimentos clínicos e cirúrgicos. O longo período até primeiro uso de procedimentos clínicos (mediana de 578 [0–1.068] dias) e cirúrgicos (mediana de 213 [30–1.034] dias) indica que esses procedimentos necessitam ainda da conclusão de ações para que possam ser utilizadas. Entre as possíveis ações estão a publicação de PCDT, adequação institucional, negociações de preço, ajustes orçamentários e estabelecimento de contratos. Embora não esteja claro o momento em que cada uma dessas ações ocorre (se antes ou após à inclusão no SIGTAP), sabe-se que ocorrem após a decisão de incorporação, o que permite a análise de alguns fatores possivelmente relacionados ao *período pós-incorporação* como um todo.

Verificou-se variação nos dias transcorridos durante o *período pós-incorporação* (período entre a decisão de incorporação e primeiro uso) para os grupos de tecnologias avaliadas. Os procedimentos cirúrgicos apresentaram o maior período (mediana de 802 [544–1.239]), e uma diferença significativamente maior foi observada na comparação de qualquer um dos procedimentos em relação às OPMs. Essas diferenças observadas entre os tipos de tecnologias podem estar relacionadas a variação de complexidade entre elas, enquanto entre os OPMs estão insumos de uso individual para cuidado em saúde, procedimentos médicos envolvem ações sobre o paciente, exigindo, frequentemente, conhecimento técnico especializado e infraestrutura específica^14,15^.

Além do grupo de organização do SIGTAP, os resultados obtidos indicam que a indicação quanto a ser necessário adequação para implementação da tecnologia no relatório Conitec, assim como ter a necessidade de adequação e o atraso de avaliação para decisão da incorporação influenciam no *período pós-incorporação*. Verificou-se período significativamente maior para as tecnologias em que não é mencionado a necessidade de adequação (*versus* ser mencionado), em que é necessária adequação (*versus* não necessitar) e para as que foi verificado atraso na incorporação (*versus* não ter atraso). Entende-se que a indicação sobre a necessidade de adequações, sejam de infraestrutura das instituições ou dos profissionais envolvidos com a tecnologia, ainda no relatório de avaliação da incorporação, é importante pois são ações que necessitam de planejamento, e se já estiverem definidas durante o processo de incorporação, possibilitam menor período na pós-incorporação. Para os casos em que há necessidade de adequação, esta pode exigir tempo considerável para finalização, visto que além do treinamento da equipe técnica responsável há uma curva de aprendizado que varia conforme a complexidade da tecnologia^15^.

Em relação ao maior período identificado para as tecnologias que já tiveram atraso na decisão de incorporação, entende-se que há complicadores da avaliação de incorporação que se estendem ao pós-incorporação. Um deles é em relação à disponibilidade de evidências científicas de qualidade, que podem influenciar tanto na decisão sobre a incorporação, como na elaboração de PCDTs, realizada no pós-incorporação^16^. Ainda, indica a complexidade de todo o processo de ATS de procedimentos e OPMs, pois tratam-se de tecnologias com características específicas, que diferem da avaliação de medicamentos^17,18^.

A fim de melhorar o processo de efetivação de tecnologias, algumas recomendações de mudança vêm sendo apontadas, entre elas, sobre a pactuação das responsabilidades de financiamento e de aquisição da tecnologia, na verificação de requisitos estruturais para a implementação de uso da tecnologia e na elaboração/atualização das diretrizes clínicas^9^, sendo que os dados deste estudo em relação às variáveis de adequação corroboram a relevância de se prever antecipadamente os requisitos para uso da tecnologia.

Entre os relatórios de incorporação analisados, 14 não tiveram identificadas as tecnologias e os códigos correspondentes no SIGTAP, sendo que 53% dessas tecnologias foram incorporadas a partir de 2020. Isso indica que, embora aprovadas para incorporação, essas tecnologias ainda não foram disponibilizadas de forma regular no SUS, ou seja, não estão ofertadas à população. Essas tecnologias não foram incluídas na análise deste estudo, mas se incluídas, aumentariam para 70% a proporção de procedimentos e OPMs em atraso.

Como limitações deste trabalho, apontamos a utilização apenas de fontes de dados disponíveis publicamente de forma online, e referentes a organização nacional para acesso às tecnologias, sem levar em conta particularidades locais entre estados e municípios. Considera-se, ainda, que por não haver transparência e previsibilidade de fluxo das atividades para o processo pós-incorporação das tecnologias analisadas, a discussão sobre cada período tem limitações. Aqui consideramos que a criação do código SIGTAP é uma etapa final do processo que permitirá o uso da tecnologia e, por esse motivo, o *período para codificação* foi definido como marco da efetivação da oferta da tecnologia. A legislação brasileira, por meio do Decreto 7.646/2011, define que uma tecnologia incorporada deve ser ofertada à população em até 180 dias, porém não define qual o marco que indicará essa oferta^19^. Entendemos que a existência do código não garante que todas as etapas do processo pós-incorporação estejam finalizadas, sendo, inclusive, verificado pela diferença entre o *período para codificação* e o *período pós-incorporação*. No entanto, considerar outro marco, como a data da primeira utilização para verificar o cumprimento do prazo legal, traz questões como a necessidade de uso para que a tecnologia seja utilizada, o que pode não representar o período em que ela já estava disponível, mas não precisou ser utilizada. Considerando esses fatores, decidimos pela escolha do menor período possível de ser medido para verificar o cumprimento do prazo.

A mensuração dos períodos e da proporção de procedimentos e OPMs não ofertadas dentro do prazo legal é apresentada neste estudo como um primeiro passo para compreensão do atual cenário para acessibilidade dessas tecnologias no país, necessário para que melhores resultados possam ser alcançados em termos de eficiência na pós-incorporação^20^. Entende-se que a agilidade na efetivação da oferta de novas tecnologias é relevante tanto para os pacientes como para os sistemas de saúde e que uma melhor compreensão do processo pós-incorporação pode impulsionar ações de aprimoramento.
